# Risk detection and assessment in small-scale metalworking industries of southwest Ethiopia

**DOI:** 10.1016/j.heliyon.2024.e39646

**Published:** 2024-10-24

**Authors:** Tariku Neme Afata, Zakariya Usmael, Megersa Werku, Tadicha Bute, Mohammedgezali Ibrahim, Debela Hinsermu

**Affiliations:** a Department of Environmental Health Science and Technology, Jimma University, Ethiopia; b Dambi Dollo Teachers College, Oromia region, Ethiopia; c Department of Public Health, Dambi Dollo University, Dambi Dollo, Ethiopia; d Department of Health Science, Bule Hora University, Bule Hora, Ethiopia

**Keywords:** Ethiopia, Metalwork industries, Risk analysis, Risk detection, Risk assessment

## Abstract

**Background:**

Workplace accidents present a significant challenge in the number of risks, working environment, and the number of workers exposed to them. These risk factors require systematic management, assessment, and control. Therefore, the present study aims to assess risk detection and assessment in small-scale metalwork industries in Jimma City, southwest Ethiopia."

**Methods:**

Data collection from two hundred employers and managers was conducted using a cross-sectional study design, incorporating a worksheet, observation, risk-detecting checklists, and technical documents. Finally, the percentage, frequency, and chi-square tests were utilized to analyze the risk factors identified in small-scale metalworking industries, and independent variables that showed a significant correlation with each dependent variable at p < 0.05 were selected for further analysis.

**Results:**

The findings showed, 1460 risks at a workshop, out of which 6.85 % were first rank, 2.74 % second rank, 73.97 % third rank, and 16.44 % were fourth rank. The most hazardous units were metal part assembly, handling, cutting, and electrical welding. Moreover, factors like heat, electric shock from the machine, noise, and vibration have an association with the occurrence of physical risk factors among the participants. Furthermore, excessive force, lifting and carrying heavy weight by hand, uncomfortable hand tools, and repetitive activity were associated with the incidence of moderate ergonomic risk factors. In addition, exposure to spraying mists, and explosion of gases and liquids under pressurized chemicals causes moderate incidence among participants. Finally, lack of experience and skills, poor work relations with colleagues and supervisors, lack of concentration, work overcapacity, and lack of training were causes of psychosocial risk factors among the participants.

**Conclusions:**

The findings reveal a multitude of risks across various ranks, with factors such as lack of knowledge, negligence of safety measures, and stress emerging as significant contributors. Addressing these issues through comprehensive safety protocols and training initiatives is paramount to ensuring the well-being of workers and the effective management of workplace risks.

## 1 Introduction

Workplace accidents present a significant challenge for retaining skilled labor, financial stability, and productivity, thus posing a threat to national progress and development [1]. Furthermore, statistics globally highlight workplace accidents as a leading cause of fatalities [2,3], imposing health, economic, and societal burdens [4,5]. While occupational accidents are inevitable, mitigating their occurrence is imperative [4,6]. However, effectively controlling accidents remains a key challenge for most managers [7]. By identifying their root causes and contributing factors, accidents can be managed more effectively [8]. Consequently, organizations often require systems to assess their operations, procedures, and the elements influencing accidents [9].

One crucial component of such systems is risk assessment, which aims to identify, evaluate, and manage factors that could compromise employee health and safety [10]. According to OHSAS18001 standards, risk assessment focuses on workplace risk factors and considers control options to determine acceptable risks [11]. Given the rising use of hazardous chemicals like heavy metals in metal industries and their adverse health effects [12,13], conducting health risk assessments becomes imperative. These assessments, conducted globally, reveal the detrimental health impacts of various chemicals [15]. For instance, welding-related fumes and gases pose significant health risks, including exposure to O_3_, NO_2_, and chromium fumes [14].

In addition to chemical risk factors, workers in metal industries are exposed to various physical risks, such as noise, mine gases, heat, poor visibility, and dust [15]. Without adequate ear protection, noise-induced hearing loss becomes a significant risk [[16], [17], [18]], compromising both worker safety and productivity [[19], [20], [21]]. Physical workload, a common risk factor, increases the likelihood of injuries, fatigue, and accidents [[22], [23], [24], [25]]. Research in metalworking industries underscores the prevalence of physical and psychosocial risk factors [26], particularly in small-scale metal industries where workers face heightened injury risks [27]. Musculoskeletal disorders (MSDs), a common consequence of repetitive tasks and heavy lifting, pose significant risks to workers' health and productivity [[28], [29], [30]]. Additionally, older age groups and gender are additional risk factors for MSDs [[31], [32], [33], [34]].

The small-scale metalworking industry significantly influences the socio-economic fabric of southwest Ethiopia by fostering employment, bolstering local development, and stimulating industrial progress. Nevertheless, inherent in its importance are occupational risk factors and risks that demand meticulous detection and assessment to safeguard the welfare of workers and the surrounding environment.

Despite its importance, research focusing specifically on risk detection and assessment within small-scale metalworking industries in southwest Ethiopia remains limited. For instance, research by Ref. [35] examines occupational health challenges in small-scale manufacturing enterprises in Addis Ababa, it does not explore the specific risks prevalent in metalworking industries.

Thus, assessing and identifying high-risk areas in metalworking industries is essential for accident prevention in Jimma, southwest Ethiopia. Therefore, this study aims to evaluate risk factors and their severity in small-scale metalworking industries, focusing on mechanical, ergonomic, chemical, and psychosocial risks. By addressing these risks, organizations can enhance workplace safety and protect workers' well-being. Moreover, this research contributes to filling the gap in risk detection and assessment practices in small-scale metalworking industries in southwest Ethiopia, paving the way for tailored risk management strategies and improved occupational safety.

## 2 Materials and methods

### 2.1 Study area

**Fig. 1:**
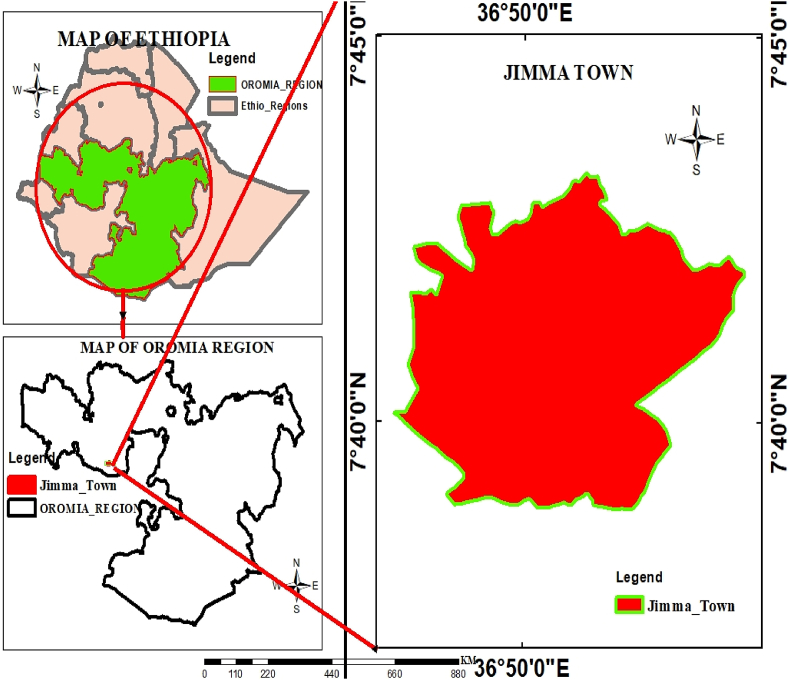
Geographical location of the study area for Jimma Town southeastern Ethiopia.

The study was conducted in Jimma, Oromia, western Ethiopia, in June 2022. Jimma, one of the zones in the Oromia Region, lies 335 km and 1760 m above sea level southwest of Addis Abeba. The town has a 44.8 square kilometers area. There are believed to be 121805 people overall, with 61453 men and 60352 women [36]. Oromo and Amhara were the two main ethnic groups in the study area. Finally, the map of the study area indicated in Fig. 1.

### 2.2 Study design

A cross-sectional design was conducted in risk detection and assessment of small-scale metalwork industries of Jimma City, western Ethiopia.

### 2.3 Source and study population

All employees working in small-scale metalwork industries located in Jimma City were the source population. Employees who were on duty in small-scale metal industries during the data collection period were the study population.

### 2.4 Sample size and sampling techniques

Single population proportion formula was used to determine the sample size for the prevalence of occupational injury with the following assumptions: p (prevalence of occupational injury among small scale metal working industries was 18 % [37] with a 95 % confidence interval, and a 5 % margin of error. By the formula:

(Equation 1) $$n=\frac{{\left(Z\alpha /2\right)}^{2}\left(p\right)\left(q\right)}{{\left(\text{SE}\right)}^{2}}$$

where?

zα/2 = 1.96

p = 0.18

q = 1− p = 1–0.18.

SE = 0.05.

Substituting these values into Equation 1

(Equation 2) $$n=\frac{{\left(1.96\right)}^{2}\left(0.18\right)\left(1‐0.18\right)}{{\left(0.05\right)}^{2}}=226$$

The overall sample size was 249 workers, accounting for a 5 % addition to the highest sample size to compensate for the non-response rate, as calculated using Equation (2). The stratified sampling method was used to collect samples from all the metal sectors of the small-scale metalworking industries in Jimma City. This method was chosen to eliminate bias when selecting samples from different population areas. First, the small metal businesses in Jimma City (the sampling frame or research population) were sorted into ten subgroups based on their physical proximity to one another. These groups consisted of Gebre, Sabaif, Awel Biya, Imiran Jamal, Tijani, Eyob and Kalkidan, Aman, Kalkidan Jibril, Asaye, and Abdu metalworking shops. Second, quotas were assigned to each subdivision of the small-scale metalwork sectors based on the number of workers. Finally, using simple random selection techniques, the study's sample was chosen from each subgroup.

### 2.5 Inclusion and exclusion criteria

All those people who do not work in the small-scale metalwork industries and are not willing to participate in the study were excluded from being included.

### 2.6 Data collection techniques

A standardized questionnaire was used to collect data from respondents at metalwork centers, administered in person. Initially prepared in English, it was then translated into Afan Oromo for clarity and back-translated into English to ensure accuracy. Both data collectors and supervisors underwent a one-day training session, with supervisors conducting daily assessments and checks of completed questionnaires for accuracy. They provided feedback to data collectors the following morning. The questionnaire was developed based on existing literature and input from study participants to investigate mechanical, ergonomic, chemical, and psychosocial risk factors in small-scale metalworking industries [[38], [39], [40], [41]]. Finally, information from two hundred small metalwork industry owners, managers, employees/workers. Additionally, observation, interviews, risk-detecting checklists, and technical documentation available in the organization were used. It also generates a list of suitable measures to reduce the risks.

### 2.7 Data processing and analysis

The collected data were defined and entered SPSS software version 24.0 (IM Corp., Armonk, NY, US). Then we used the percentage, frequency, and chi-square tests to examine hazard severity and associated risk factors of small-scale metal industries. Finally, only elements exhibiting a significant correlation (*p* < 0.05) with each dependent variable were chosen and analyzed. Finally, we used bivariate and multivariable analyses to look at the factors that have an association with dependent variables. Only factors exhibiting a significant correlation (*p* < 0.25) with each dependent variable in the bivariate analysis were chosen and adjusted in the multivariate logistic regression analysis. All variables with a p-value of <0.05 were considered statistically significant associations with micronutrient deficiencies.

### 2.8 Operational definitions

Hazard: A hazard is anything with the potential to cause harm to health or safety, or damage to property, equipment, or the environment.

Risk: Risk is the probability that a hazard will cause injury, illness, or damage to property, equipment, or the environment, along with the potential severity and long-term consequences.

**Small-scale business:** Any industry that uses power-driven machines and employs less than 10 workers [42].

**Severity of injury:** The extent of harm or damage that can result from exposure to identified hazards. This is a critical component of risk assessment and management, as it helps prioritize risks and determine the necessary control measures [43].

**Work-related injury:** A work-related injury is any physical harm or illness that occurs because of performing job duties or being present at the workplace. These injuries can vary widely in severity and type, ranging from minor to severe accidents that result in long-term disability or even death [44].

## 3 Results

### 3.1 Socio-demographic characteristics of respondents

Among the two hundred respondents studied the average age was 27.3 ± 7.36 years. Approximately 57.5 % of workers were aged between 15 and 53 years. Males constituted 69.5 % of the workforce, outnumbering females at 30.5 %. Marital status indicated that 104 workers (52 %) were married. About 20.5 % of respondents had no formal education, while nearly half (48.5 %) had completed primary school. Most respondents (56 %) were Muslim. In terms of work experience, 121 respondents (60.5 %) had less than five years of metalwork activities.

Additionally, the analysis of the chi-square (χ^2^) values reveals significant associations between certain explanatory variables and levels of risk factors. Age emerges as a significant variable, especially for individuals under 25 years, who show a significant association with both high and very low-risk factors (χ^2^ = 0.01). This indicates that younger individuals are more likely to experience extreme levels of risk. Sex also plays a role, with males showing a significant association with moderate risk factors (χ^2^ = 0.01), which suggests that males are notably more likely to experience moderate risk levels.

For education, individuals with no formal education exhibit significant associations with high (χ^2^ = 0.01), moderate (χ^2^ = 0.01), low (χ^2^ = 0.01), and very low-risk factors (χ^2^ = 0.01), indicating that lack of formal education is strongly linked to varying risk levels. Finally, working experience shows that individuals with five years or less of experience have a significant association with moderate (χ^2^ = 0.04) and low-risk factors (χ^2^ = 0.02), suggesting that less experienced individuals are more likely to encounter these risk levels.

### 3.2 Risk ranking based on the severity of the risk and frequency of the risk on jobs

The analysis of risk severity and frequency across various jobs in the small-scale metalworking industries provides significant insights into the distribution and intensity of occupational risk factors. Metal part assembly, identified as one of the most hazardous activities with 180 total risks, featured twenty risks at the highest severity level (1), none at rank 2, 120 at rank three, and sixty at rank four. In contrast, rinsing, which had forty risks in total, all ranked at the lowest severity level (4). Handling tasks accounted for two hundred identified risks, evenly distributed across severity levels: twenty at rank 1, 20 at rank 2, 140 at rank three, and twenty at rank four (see Table 1).

Cutting, another high-risk activity with two hundred risks, included forty at rank 1, 20 at rank 2, 140 at rank three, and twenty at rank four. Bending tasks encompassed two hundred risks, primarily at moderate to low severity levels, specifically 160 at rank 3 and 40 at rank four. Drilling activities, totaling 120 risks, were exclusively ranked at level 3. Drawing on a sheet, like drilling, had sixty risks all ranked at level 3, indicating moderate risk factors. Electrical welding, which featured 180 risks, showed 20 at rank 1 and 160 at rank 3, highlighting significant high and moderate hazard involvement without risks at ranks 2 and 4. Finally, CO_2_ welding, associated with 80 risks, all ranked at level 3 (Table 2).

**Table 1:** Association of sociodemographic characteristics with risk factors in the small metalworking industries of Jimma City, Ethiopia.

Explanatory Variables		f (%)	Level of risk factors											
			High			Moderate			Low			Very low		
			Yes	No	χ2	Yes	No	χ2	Yes	No	χ2	Yes	No	χ2
Age in years	<25	70(35)	35	35	0.01∗	58	12	0.7	44	26	0.96	49	21	0.01∗
	25–35	115(57.5)	92	23		87	28		70	45		86	29	
	>35	15(7.5)	13	2		15	0		9	6		2	13	
Sex	Male	139(69.5)	97	42	0.92	104	35	0.01∗	85	54	0.88	98	41	0.36
	Female	61(30.5)	43	18		56	5		38	23		39	22	
Ethnicity	Oromo	169(84.5)	123	46	0.01∗	129	40	0.01∗	106	63	0.7	126	43	0.01∗
	Amhara	26(13)	12	14		26	0		14	12		8	18	
	Others	5(2.5)	5	0		5	0		3	2		3	2	
Religion	Orthodox	43(21.5)	30	13	0.82	31	12	0.02∗	33	10	0.1	28	15	0.04∗
	Muslim	112(56)	77	35		84	28		65	47		85	27	
	Catholic	7(3.5)	6	1		7	0		5	2		3	4	
	Protestant	38(19)	27	11		38	0		20	18		21	17	
Marital	Married	104(52)	80	24	0.13	152	36	0.02∗	115	73	0.67	129	59	0.6
	Single	88(44)	56	32		4	0		2	2		2	2	
	Divorced	6(3)	4	2		2	4		4	2		4	2	
	Windowed	2(1)	0	2		2	0		2	0		2	0	
Education	No formal Educ.	41(20.5)	20	21	0.01∗	35	6	0.01∗	31	10	0.01∗	21	20	0.01∗
	Primary school	97(48.5)	73	24		69	28		65	32		75	22	
	Secondary school& above	62(31)	47	15		56	6		27	35		41	21	
Working experience	≤5 Years	121(60.5)	85	36	0.93	91	30	0.04∗	82	39	0.02∗	77	44	0.07
	>5 Years	79 (39.5)	55	24		69	10		41	38		60	19	

Abbreviations (χ2: Chi square, ∗: Significant value, f: frequency).

**Table 2:** Risks ranking based on jobs for small metalworking industries of Jimma City, Ethiopia.

The severity of the risk	Risk frequency based on risk ranking and jobs				Total
	1	2	3	4	
Metal part assembly	20	0	120	60	180
Rinsing	0	0	0	40	40
Painting	0	0	100	60	160
Handling	20	20	140	20	200
Cutting	40	20	140	20	200
Bending	0	0	160	40	200
Drilling	0	0	120	0	120
Drawing on a sheet	0	0	60	0	60
Electrical welding	20	0	160	0	180
CO_2_ welding	0	0	80	0	80

**Fig. 2:**
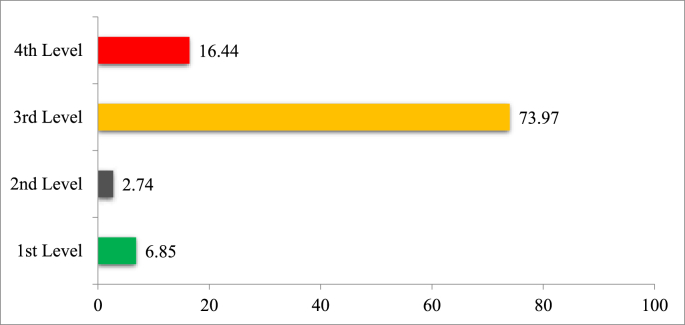
Percentage of risks based on their ranking for small metalworking industries of Jimma City, Ethiopia.

This study showed that 6.85 % of the risks were 1st rank (high), 2.74 % were 2nd rank (moderate), 73.97 % were 3rd rank (low), and 16.44 % were 4th rank (very low) (Fig. 2). The results also showed that the third risks were the largest group of risks and controlling these risks would be a great step toward reducing accidents and damages.

### 3.3 Characteristics of the risk factors

The table summarizes the association between various sources of risk factors and their levels in small-scale metalworking industries in Jimma City, southwest Ethiopia. It categorizes the risk factors into physical, ergonomic, mechanical, chemical, and psychosocial. The number of occurrences was indicated by high, moderate, low, and very low levels, along with the chi-square (χ2) values to denote statistical significance. Among the physical risk factors, heat stress shows significant associations at moderate (χ2 = 0.02∗), low (χ2 = 0.03∗), and very low (χ2 = 0.01∗) levels.

In the ergonomic category, excessive forces show a significant association at the moderate level (χ2 = 0.03∗), and for mechanical risk factors, ungraded moving parts of the machine show a significant association at the low level (χ2 = 0.04∗). Chemical risk factors, specifically exposure to inorganic welding fumes, exhibit significant associations at both moderate (χ2 = 0.01∗) and low levels (χ2 = 0.02∗), while among the psychosocial risk factors, lack of enough experience and skills shows a significant association at the low level (χ2 = 0.03∗) (Table 3).

**Table 3:** Association of the source of risk factors and their levels in small-scale metalworking industries of Jimma City, southwest Ethiopia.

Source of the risk factors		Level of risk factors											
		High			Moderate			Low			Very low		
		Yes	No	χ2	Yes	No	χ2	Yes	No	χ2	Yes	No	χ2
Physical	Heat stress	41	20	0.79	54	7	0.02∗	37	24	0.03∗	37	24	0.01∗
	Electric shock from the machine	21	9		29	1		14	16		21	9	
	Prolonged exposure to high levels of noise	46	21		49	18		50	17		42	25	
	Hand-arm vibration syndrome	32	10		28	14		22	20		37	5	
Ergonomic	Excessive forces	42	23	0.32	44	21	0.03∗	42	23	0.89	46	19	0.44
	Lifting and carrying heavy weight by hand	34	18		45	7		30	22		33	19	
	Uncomfortable hand tools	28	9		32	5		23	14		23	14	
	Repetitive Strain Injuries	36	10		39	7		28	18		35	11	
Mechanical	Ungraded moving parts of the machine	50	14	0.38	52	12	0.98	42	22	0.04∗	43	21	0.91
	Not working with procedure	38	18		44	12		30	26		40	16	
	Slippery floors	26	14		32	8		31	9		28	12	
	improper designs ladders	26	14		32	8		20	20		26	14	
Chemical	Exposure to inorganic welding fumes	55	26	0.71	56	25	0.01∗	56	25	0.02∗	55	26	0.88
	Exposure to spraying mists	37	17		48	6		25	29		36	18	
	Explosion of gases and liquids under pressure	48	17		56	9		42	23		46	19	
Psychosocial	Lack of enough experience and skills	43	17	0.44	48	12	0.91	42	18	0.03∗	40	20	0.62
	Poor work relations with colleagues and supervisors	13	7		16	4		10	10		13	7	
	Remuneration and annual leave issues	12	8		16	4		15	5		15	5	
	Sexual harassment and lack of concentration	14	6		16	4		11	9		13	7	
	Work over capacity	28	12		32	8		21	19		26	14	
	Lack of promotion and training	30	10		32	8		24	16		30	10	

Abbreviations (χ2: Chi square, ∗: Significant value).

## 4 Discussion

The analysis identified unique risks that are present in small-scale metal industries at work areas/departments, units, facilities, and activities/functions. From our findings for small-scale metalworking industries, the most hazardous units were metal part assembly, handling, cutting, and electrical welding. The lowest hazardous units were metal part assembly, rinsing, painting, cutting, and bending. According to the literature review, the main causes of these risks are lack of knowledge about safe ways of doing jobs (skills), negligence of safety measures (attitude), beliefs that doing jobs in an unsafe manner would not cause a problem (belief), failure to observe safety measure due to stresses (emotional), and thinking that safety measures are time-consuming (personality) [45]. Thus, taking into account the type of detected risks, designing and implementing preventive repair services would be effective in controlling detected risks [46,47].

Moreover, factors like heat, electric shock from the machine, noise, and vibration have an association with the occurrence of physical risk among the participants. These physical risks that occur on small-scale metal workers explained the largest percentage of variance for adverse safety events and accidents across all of the industries sampled [48,49]. The physical risks such as noise, heat, dust, chemicals, and hazardous tools and equipment were related to employee involvement in safety activities [50,51]. The use of insecure and faulty equipment also generates excessive noise and exhales dust particles into the small-scale metalwork industries [52,53]. All these factors can impose upon workers' safety experience by obstructing workers’ ability to hear and understand safety signals.

Furthermore, excessive forces, lifting and carrying heavy weight by hand, uncomfortable hand tools, and repetitive activity have an association with the occurrence of moderate ergonomic risk factors among the participants. Musculoskeletal risks at work are typically caused by physical strain and awkward work postures such as lifting, strong gripping, twisting, bending, and kneeling [54,55]. These are the basic work postures that may result in musculoskeletal illnesses [56,57]. The assembling of metal parts, painting, handling, cutting, bending, drilling, electrical welding, and drawing on a sheet are all manual tasks that must be done repeatedly while slouching or twisting for an extended period [58,59]. Additionally, a variety of other elements, such as work structure, psychosocial factors, tools, personality differences, and the workplace itself, might contribute to complaints in addition to the working environment.

In addition, inorganic welding fumes, spraying mists, gases, and liquid chemicals have an association with the occurrence of moderate risk among the participants. The current investigation found that metal vapors posed the greatest risk of chemical exposure in small-scale metalworking. Small-scale metalwork enterprises, which are frequently run by a small number of people, expose their workers to more dangerous conditions than those in other industries, such as exposure to metal vapors [40,60]. According to this study, small-scale metalworkers are more likely to be exposed to respiratory contaminants. The levels of chemicals present at work have a significant impact on estimating the risk of chemical exposure [40]. Furthermore, long working hours and a lack of ventilation systems to lower the risk ranking of exposed workers were the lowest [60].

Finally, lack of enough experience and skills, poor work relations with colleagues and supervisors, remuneration and annual leave issues, sexual harassment and lack of concentration, work overcapacity, and lack of promotion and training have an association with the occurrence of psychosocial risk factors among the participants [61]. The psychological factors that lead to risky circumstances in a certain work environment. In particular, some study shows that certain actions can lead to unwanted subsequent outcomes such as accidents or injuries [60] and different accidents and injuries reported in the small metal industries sector are thought to be caused by psychosocial risks [62]. In addition, witnessed accidents were associated with ambient conditions in the small-scale metalwork industries [63].

This study's main flaw was improper employer and employee engagement, which kept the true data from data collectors. Employee engagement is a multi-faceted construct (cognition, emotions, and behaviors) that was found to have a positive relationship with individual performance (organizational commitment, positive behavior, etc.) in metalworking industries. Finally, the study has certain limitations. Respondents may not remember instances of occupational injury that occurred over a year ago, which could lead to an underestimation of the overall prevalence (recall bias). Additionally, study participants might believe that reporting an injury would be advantageous, potentially leading to an overestimation of prevalence (social desirability bias). Moreover, comparing data was challenging due to the lack of similar research, particularly in Ethiopia.

## 5 Conclusion

These findings identified about 1460 risks, with varying degrees of severity: 6.85 % classified as first rank, 2.74 % as second rank, 73.97 % as third rank, and 16.44 % as fourth rank. Metal part assembly, handling, cutting, and electrical welding emerged as the most hazardous activities. Furthermore, physical risk factors were associated with heat, electric shock, noise, and vibration. Additionally, moderate ergonomic risk factors were linked to excessive forces, heavy lifting, uncomfortable tools, and repetitive tasks. Moreover, inorganic welding fumes, mists, gases, and liquid chemicals were related to moderate and low risks. Finally, psychosocial risk factors were attributed to factors including lack of experience, poor work relations, concentration issues, work overcapacity, and inadequate training need comprehensive interventions to mitigate these risks and ensure the safety and well-being of workers. By implementing enhanced risk management strategies, ergonomic improvements, chemical exposure controls, and psychosocial support initiatives, stakeholders can create safer and healthier work environments for employees in the region.

## CRediT authorship contribution statement

**Tariku Neme Afata:** Writing – review & editing, Writing – original draft, Software, Resources, Methodology, Formal analysis, Data curation, Conceptualization, Investigation, Supervision, Validation, Visualization. **Zakariya Usmael:** Writing – review & editing, Supervision, Conceptualization, Data curation, Formal analysis, Methodology. **Megersa Werku:** Writing – review & editing, Supervision, Conceptualization, Data curation, Formal analysis, Funding acquisition, Methodology, Resources, Writing – original draft. **Tadicha Bute:** Writing – review & editing, Methodology, Resources, Writing – original draft. **Mohammedgezali Ibrahim:** Conceptualization, Data curation, Formal analysis, Writing – review & editing. **Debela Hinsermu:** Conceptualization, Data curation, Formal analysis, Methodology, Validation, Visualization, Writing – review & editing.

## Ethical standards disclosure

The ethical approval to conduct this research was obtained from the Institute of Health Review Board (IRB) of Jimma University, southwest Ethiopia, on 18/10/2019 (No. IHRPGD/407/2019), and written informed consent was obtained from the study participants. All subjects participated in the study voluntarily.

## Consent for publication

Not applicable.

## Availability of data and materials

The datasets used during the current study are available in the manuscript and supplementary materials.

## Declaration of competing interest

The authors declare that they have no known competing financial interests or personal relationships that could have appeared to influence the work reported in this paper.
